# Effect of Mineral Element Imbalance on Neutrophil Respiratory Burst Function and Inflammatory and Antioxidant Responses in Sheep

**DOI:** 10.3390/vetsci10040241

**Published:** 2023-03-23

**Authors:** Weiqi Liu, Di Wang, Qijun Zhou, Jianfa Wang, Shuai Lian

**Affiliations:** 1 State Key Laboratory of Animal Nutrition, College of Animal Science and Technology, China Agricultural University, Beijing 100193, China; 2 College of Animal Science and Veterinary Medicine, Heilongjiang Bayi Agricultural University, Daqing 163319, China

**Keywords:** sheep, mineral elements, neutrophils, respiratory burst function

## Abstract

**Simple Summary:**

The imbalance of mineral elements affects cellular immunity, organismal inflammation and antioxidant function. This study established a model of mineral element homeostatic imbalance to assess the impact on these functions in sheep. The results showed that mineral element imbalance enhances the respiratory burst function of neutrophils and alters the status of inflammatory and antioxidant responses in sheep.

**Abstract:**

This study established a model of mineral element homeostatic imbalance and examined the respiratory burst function of peripheral blood neutrophils and inflammatory and antioxidant indicators before and after the imbalance in sheep. The results showed that after an EDTA injection, the number of activated neutrophils in the peripheral blood was significantly higher than that in the control group (*p* < 0.01). In addition, the serum IL-6 level was significantly increased (*p* < 0.05) and matrix metalloproteinase 7 (MMP7) was inhibited (*p* < 0.05), but returned to a normal level one week after the injection. Tissue inhibitor of metalloproteinase 1 (TIMP1) levels were consistently higher after the injection and significantly higher than in the control group (*p* < 0.05). CuZn-SOD, TNOS activity, serum creatinine and urea nitrogen levels were significantly higher than before the injection (*p* < 0.05). Combining the results of previous studies, the EDTA injection altered the metabolism and transcription of peripheral blood neutrophils. These changes enhance the respiratory burst function of neutrophils and alter the status of inflammatory and antioxidant indicators such as IL-6 and CuZn-SOD.

## 1. Introduction

Neutrophils are the first line of natural immune defense against external pathogen invasion in healthy ruminants such as dairy cows [1]. Under a moderate immune response, Toll-like receptors on endothelial epithelial cells recognize pathogen-associated molecules and promote the secretion of cytokines and chemokines [2]. Neutrophils are recruited to the site of infection and clear pathogenic bacteria through phagocytosis, respiratory bursts and the formation of neutrophil extracellular traps (NETs), attracting lymphocytes [3]. After this, neutrophils can revert to apoptosis, and macrophages clear the apoptotic neutrophils and secrete anti-inflammatory repair molecules to maintain local immune homeostasis, such as in the mammary gland and endometrium [4,5]. However, persistent infiltration is detrimental, as the prolonged exposure of the infected site to pro-inflammatory cytokines and reactive oxygen metabolites predisposes the individual to chronic inflammation and oxidative stress, which can lead to diseases such as subclinical endometritis and infertility [5]. Mineral elements are key components of the antioxidant system, metabolism, protein synthesis pathways and membrane integrity [6]. Postpartum mineral supplementation in animals is beneficial to the formation of superoxides [7]; when the need for mineral elements increases before and after delivery, the concentration of trace elements in the blood and liver decreases. The supplementation of amino acid complexes of zinc (Zn), manganese (Mn), copper (Cu) and cobalt glucose enanthate during the perinatal period can improve the function of neutrophils, endow them with a stronger ability to defend against pathogen invasion and promote rapid uterine recovery during early lactation, while also improving liver function and productivity [8].

Mineral elements are indispensable nutrients for animals to maintain life and production. They participate in a series of physiological and biochemical processes in animals and play an important role in animal growth, reproduction and immune defense. However, the negative effects of adding mineral elements require further research. In livestock and poultry production, the non-standard use of mineral additives is common. The synergistic and antagonistic interaction between mineral elements [9,10], and the metabolic level and physiological function of ruminants, change significantly under different feeding conditions or in special growth periods. As a consequence, a homeostasis imbalance and immune dysfunction can often occur. Therefore, mineral element homeostasis imbalance may affect animal inflammation, antioxidant capacity and neutrophil function, predisposing them to chronic inflammation and oxidative stress.

In the early stage of this research, we selected sheep as the object of study and established a model of mineral element homeostasis imbalance by using an intravenous injection of EDTA. As a metal chelator, EDTA could complex with calcium (Ca), Cu, Zn and other mineral elements, and, based on the synergistic and reciprocal effect between elements, EDTA injection could cause the imbalance of mineral elements in animals. This model demonstrated that mineral element homeostasis imbalance affected the metabolic process through the signaling pathways of amino acid metabolism, lipid metabolism and nucleotide metabolism of neutrophils [11], and changed the gene transcription level of neutrophils [12]. However, whether these changes are involved in the inflammatory response, antioxidant response and respiratory burst function of neutrophils is unclear. In this study, blood gas and electrolyte parameters (Na^+^, K^+^ and Cl^−^, etc.), neutrophil respiratory burst and inflammatory (IL-6, IL-1β and iNOS, etc.) and antioxidant parameters (CuZn-SOD, GSH-PX, etc.) were measured to address these issues.

## 2. Materials and Methods

### 2.1. Animals and Experimental Design

**Figure 1:**
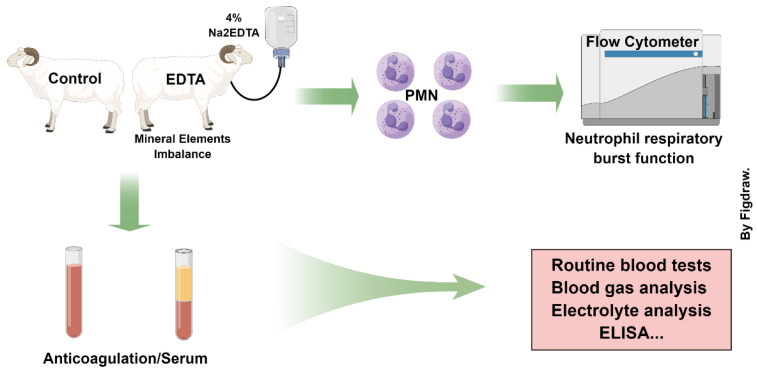
Experimental grouping and treatment.

Ten healthy sheep weighing 30 kg were pre-fed for two weeks before the formal experiment began. A central venous catheter was installed in 10 sheep, the blood was collected 24 h after it was installed and used as the control group (CON), and then EDTA was injected intravenously to construct a model of mineral element homeostasis imbalance in sheep and set as the experimental group (EDTA) (Figure 1). Seven days after the injection, blood samples were collected and designated as the recovery group (HF). For the specific operation procedure and experimental design, please refer to the preliminary research of the laboratory [11,12]. The experiment was approved by the Animal Care Committee of Heilongjiang Bayi Agricultural University.

### 2.2. Sample Collection

Whole blood samples of sheep before and after the EDTA injection were collected via central venous catheters. Serum and neutrophils were separated (the specific collection method referred to the laboratory’s preliminary research [12]) and used in electrolyte detection, flow cytometry analysis, ELISA and other experiments.

### 2.3. Routine Blood Tests

EDTA anticoagulated blood was collected 24 h before and 24 h after the installation of the central venous catheter, and routine blood tests were performed by using a dry blood cell analyzer (VetAutoread, IDEXX, Westbrook, ME, USA).

### 2.4. Blood Gas and Electrolyte Analysis

First, 0.25 mL of heparin lithium anticoagulant blood was collected (arterial blood); we used the “Electrolytes Cassette” (IDEXX, Westbrook, ME, USA) and scanned the barcode, and used the correct electrolyte and blood gas analyzer (IDEXX, Westbrook, ME, USA) to determine the K^+^, Na^+^, Cl^−^, pH, PCO2 (Electrolytes Cassette, IDEXX, Westbrook, ME, USA), Ca^2+^ (Ionized Calcium Cassette, IDEXX, Westbrook, ME, USA) and other indicators in the whole blood of sheep.

### 2.5. Flow Cytometry Analysis

Neutrophil respiratory burst detection kits (abs50002, Absin, Shanghai, China) were used to detect the respiratory burst function. We added 50 μL of foponate (PMA) to 50 μL of anticoagulated blood and incubated it for 15 min at 37 °C. Then, 25 μL of dihydrorhodamine was added to each tube and incubated for 5 min at 37 °C while protected against light; we added 1 mL of diluted hemolysin (diluted 1:10 with deionized water) to each tube and they were protected against light at room temperature for 15 min. The supernatant was discarded after 2 washes with PBS, followed by centrifugation at 1500 rpm for 5 min. We added 0.5 mL of PBS to resuspend the cells and transferred them to a flow tube for detection using flow cytometry (CytoFLEX, Beckman, Brea, CA, USA).

### 2.6. ELISA, Colorimetric and Hydroxylamine Method Detection

IL-6 (SEA079Ov, Cloud-Clone, Wuhan, China), IL-1β (SEA563Ov, Cloud-Clone, Wuhan, China), TNF-α (SEA133Ov, Cloud-Clone, Wuhan, China), TIMP1 (SEA552Ov, Cloud-Clone, Wuhan, China) and MMP7 (SEA102CP, Cloud-Clone, Wuhan, China) in serum were detected by ELISA. Ceruloplasmin (CP; A029-1-1, Nanjing Jiancheng, Nanjing, China), nitric oxide synthetase (NOS; A014-1-2, Nanjing Jiancheng, Nanjing, China) and glutathione peroxidase (GSH-PX; A005-1-2, Nanjing Jiancheng, Nanjing, China) in serum were detected by colorimetric methods; superoxide dismutase (SOD; A001-2-2, Nanjing Jiancheng, Nanjing, China) and total superoxide dismutase (T-SOD; A001-1-1, Nanjing Jiancheng, Nanjing, China) in serum were detected by hydroxylamine. The procedure was performed according to the kit instructions.

### 2.7. Statistical Analysis

The one-way ANOVA multiple comparison method and GraphPad Prism software (8.0.2) were used to analyze the data of ELISA, colorimetric and hydroxylamine test indicators. The results were expressed as the mean ± standard deviation. The unpaired T test of the GraphPad Prism software (8.0.2) was used to analyze the data on the respiratory burst detection, routine blood tests, blood gas detection and ion detection. A value of *p* < 0.05 was considered statistically significant.

## 3. Results

### 3.1. Effect of Central Venous Catheters on Blood Routines in Sheep

The results of preoperative and postoperative routine blood tests are shown in Table 1. There was no significant increase in the number of white blood cells and granular white blood cells after surgery, and no significant change in the average hemoglobin concentration, red blood cell volume, lymphocyte to monocyte ratio, etc. This indicates that the central venous catheterization of the neck had no significant impact on the blood vessel and blood indexes, and there was no significant difference in the six routine blood indexes measured before and after surgery (*p* > 0.05).

### 3.2. Effect of EDTA Injection on Blood Gas and Electrolytes in Sheep

As shown in Table 2, before the injection, the electrolyte content in the blood of the sheep was at a normal level. After the EDTA injection, the Na^+^, K^+^ and Cl^−^ content in the blood of sheep in the EDTA group was significantly lower than those in the control group (*p* < 0.01), and the pH, PCO2, HCO3^−^, tCO2 and Ca^2+^ content did not change significantly (*p* > 0.05).

### 3.3. Mineral Element Homeostasis Imbalance Affects Neutrophil Respiratory Burst Function

**Figure 2:**
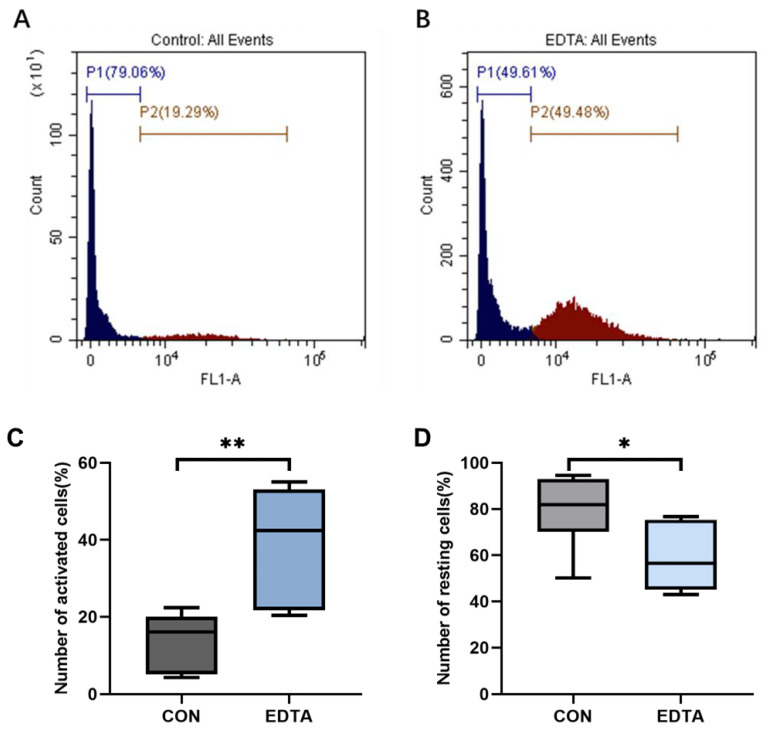
Flow cytometry of the neutrophil respiratory burst. (**A**) Flow cytometry spectrum of neutrophils in the control group. (**B**) Flow cytometry spectrum of neutrophils in the EDTA group. (**C**) The percentage of activated cells in the control and EDTA groups. (**D**) The percentage of stationary cells in the control and EDTA groups. CON: control group, EDTA: experimental group. Statistically significant differences are indicated by * *p* < 0.05, ** *p* < 0.01.

As shown in Figure 2, after the injection of EDTA, the number of activated neutrophils in the peripheral blood of sheep was significantly higher than that of the control group (Figure 2C, *p* < 0.01), and the number of neutrophils in the resting state was significantly lower (Figure 2D, *p* < 0.05).

### 3.4. Effects of Mineral Element Homeostatic Imbalance on Serum IL-6, IL-1β and TNF-α

**Figure 3:**
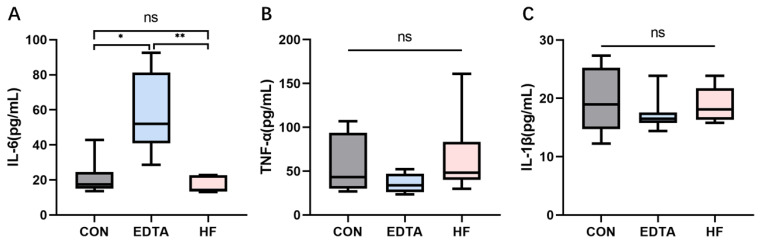
Levels of IL-6, TNF-α and IL-1β in the serum of each group detected by ELISA. (**A**) Level of IL-6 in the serum of each group. (**B**) Level of TNF-α in the serum of each group. (**C**) Level of IL-1β in the serum of each group. CON: control group, EDTA: experimental group, HF: recovery group. Statistically significant differences are indicated by * *p* < 0.05, ** *p* < 0.01.

As shown in Figure 3A, the mineral element homeostasis imbalance induced by the intravenous injection of EDTA significantly increased the serum IL-6 level (*p* < 0.05); one week after the injection, the serum IL-6 level returned to that before the injection (*p* > 0.05), which was significantly lower than that of the EDTA injection group (*p* < 0.01). In addition, as shown in Figure 3B,C, the EDTA injection did not significantly affect the levels of serum TNF-α and IL-1β (*p* > 0.05).

### 3.5. Effects of Mineral Element Homeostatic Imbalance on Serum MMP7 and TIMP1

**Figure 4:**
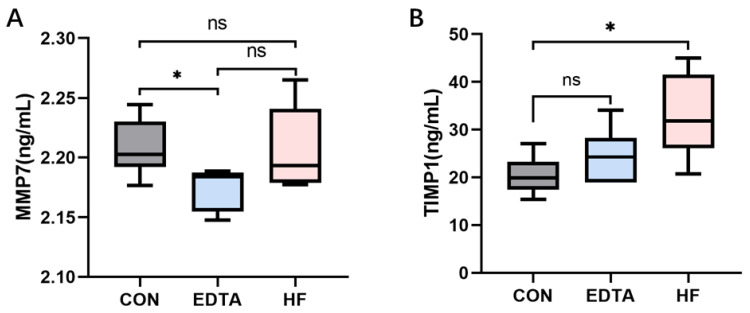
Levels of MMP7 and TIMP1 in the serum of each group detected by ELISA. (**A**) Level of MMP7 in the serum of each group. (**B**) Level of TIMP1 in the serum of each group. CON: control group, EDTA: experimental group, HF: recovery group. Statistically significant differences are indicated by * *p* < 0.05.

As shown in Figure 4A, the level of MMP7 after the EDTA injection was significantly lower than that before the injection (*p* < 0.05). One week after the injection, the level of MMP7 in the serum was recovered, and there was no significant difference compared with the control group before the injection (*p* > 0.05). The level of TIMP1 increased after the EDTA injection (Figure 4B), and there was no significant difference compared with the control group (*p* > 0.05). One week after the injection, the level of TMMP1 did not return to the normal concentration but further increased, and it was significantly higher than that before the injection (*p* < 0.05).

### 3.6. Effects of Mineral Element Homeostatic Imbalance on Serum CuZn-SOD, GH-PX, iNOS, TNOS, T-SOD and CP Vitality

**Figure 5:**
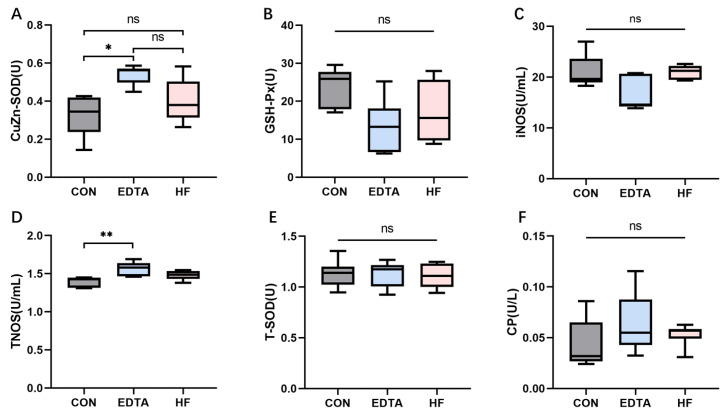
The activity of (**A**) CuZn-SOD, (**B**) GH-PX, (**C**) iNOS, (**D**) TNOS, (**E**) T-SOD and (**F**) CP in the serum of each group detected by colorimetric and hydroxylamine test methods. CON: control group, EDTA: experimental group, HF: recovery group. Statistically significant differences are indicated by * *p* < 0.05, ** *p* < 0.01.

As shown in Figure 5A, the activity of CuZn-SOD was significantly increased after the EDTA injection compared with before the injection (*p* < 0.05). One week after the injection, the activity of CuZn-SOD returned to the pre-injection level (*p* > 0.05). As shown in Figure 5B, the activity of GSH-PX decreased after the EDTA injection compared with before the injection, but the difference was not significant (*p* > 0.05). One week after the end of the injection, the activity of GSH-PX was recovered. As shown in Figure 5C,E, the activities of iNOS and T-SOD in the serum did not change significantly before or after the EDTA injection (*p* > 0.05). As shown in Figure 5D, compared with the control group, the activity of TNOS was significantly increased after the EDTA injection (*p* < 0.01) and returned to the pre-injection level one week after the injection. As shown in Figure 5F, the activity of CP after the EDTA injection was not significantly different from that before injection but slightly increased (*p* > 0.05) and recovered one week after the injection.

### 3.7. Effects of Mineral Element Homeostatic Imbalance on Serum Blood Urea Nitrogen (BUN) and Creatinine (Crea)

**Figure 6:**
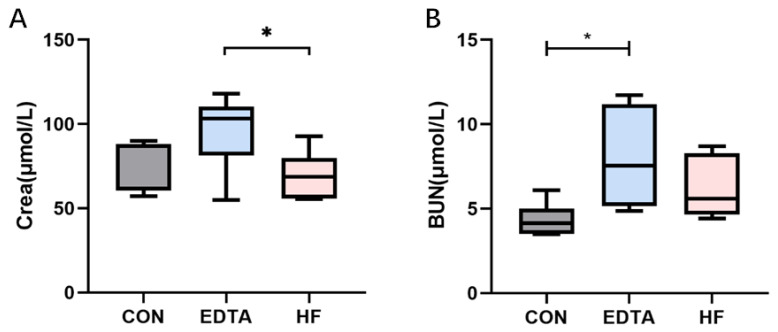
Levels of Crea and BUN in the serum of each group detected by automatic biochemical analyzer. (**A**) Level of Crea in the serum of each group. (**B**) Level of BUN in the serum of each group. CON: control group, EDTA: experimental group, HF: recovery group. Statistically significant differences are indicated by * *p* < 0.05.

As shown in Figure 6A, the serum creatinine level increased after the EDTA injection but returned to a normal level one week after the injection, and this was significantly different from the EDTA group (*p* < 0.05). As shown in Figure 6B, the level of serum urea nitrogen also increased significantly after the EDTA injection (*p* < 0.05); although it recovered a week after the injection, it was still slightly higher than the normal level, but the difference was not significant (*p* > 0.05).

## 4. Discussion

Blood gas analysis detection is an important tool to measure respiratory function and acid–base homeostasis in animals [13]. In this study, a model of mineral homeostatic imbalance in sheep was established through the central venous catheter injection of EDTA. Routine blood test and blood gas analysis results showed no effect on the routine blood parameters, and the pH of sheep in the EDTA group did not change significantly, suggesting that the injection of EDTA did not affect the blood pH in sheep. The PCO2, tCO2 and the level of HCO3^−^ in the blood of sheep in the EDTA group did not change significantly either, indicating that no respiratory or metabolic acidosis occurred in sheep after the EDTA injection. However, EDTA significantly reduced the levels of K^+^, Na^+^ and Cl^−^ compared with the control group, but they remained within the normal range [14]. It is well known that EDTA is a broad-spectrum metal ion chelator [15]; this may be due to an imbalance in electrolyte homeostasis caused by the chelation of EDTA with other metal ions.

In our previous study, an ionomic analysis of serum (performed using inductively coupled plasma mass spectrometry—ICP-MS) was used to accurately detect 53 mineral elements in sheep serum before and after an injection of EDTA, and it was confirmed that the EDTA could lead to an imbalance in mineral element homeostasis in sheep. The levels of P, Zn, K and other elements in serum were significantly decreased after EDTA injection, while the concentration of Cu was significantly increased [12]. In addition, after the injection of EDTA, the total calcium level in the blood of sheep showed a slight upward trend. One of the reasons for this result may be that the dynamic balance of calcium in sheep was broken after the EDTA injection, prompting the bones to continuously undergo osteogenesis and osteolysis to keep the total calcium in the body relatively constant [16]. Another possible reason is the mild intoxication of renal cells by EDTA, which prevents the rapid clearance of EDTA calcium complexes [15]. In this study, an increase in serum creatinine and blood urea nitrogen levels was observed after EDTA injection, which may be due to the decrease in the zinc ion concentration caused by EDTA. Zinc plays an important role in animals, and zinc deficiency can increase the concentration of ROS and inflammatory factors, leading to the dysfunction of animals and affecting the occurrence and development of diseases [17]. Zinc deficiency can also lead to a decrease in zinc content in renal tissue, renal vasodilatation and hyperemia, inflammatory cell infiltration, and an increase in creatinine and urea nitrogen levels [17]. Matrix metalloproteinases (MMPs) belong to zinc-related proteins, which are proteolytic enzymes involved in physiological processes such as tissue repair and can remodel the extracellular matrix [18]. As endogenous inhibitors, TIMPs can downregulate the activity of MMPs, and the interaction between TIMPs and MMPs can balance the synthesis and degradation of the extracellular matrix [19]. The disequilibrium between the increased expression of TIMP1 and the inhibited expression of MMP7 is likely to lead to an imbalance in the production and degradation of the extracellular matrix, which in turn promotes renal fibrosis [17]. In this experiment, after the injection of EDTA, there was an imbalance involving an MMP7 decrease and TIMP1 expression increase, so the homeostasis imbalance of zinc and other mineral elements induced by EDTA is likely to cause damage to the kidneys of ruminants. 

TIMP1 can also regulate the level of IL-6 and plays a role by activating the downstream signal transducer and activator of transcription 3 (STAT3) signaling [20]. In this experiment, the level of IL-6 was significantly increased after the injection of EDTA; in addition to being related to TIMP1, it may also be regulated by the S100 family proteins discussed in our previous studies [7]. S100 proteins could bind metal ions such as Ca^2+^, Mn^2+^, Cu^2+^, Fe^2+^, Ni^2+^ and Zn^2+^ [21], with a dual function of nutritional immunity and the activation of inflammation [22]. Previous studies in our laboratory had found that the levels of S100A8, S100A9 and S100A12 genes in the S100 family were extremely significantly increased after the injection of EDTA [12]. Studies have shown that the overexpression of S100A8 and S100A9 genes can significantly enhance phorbol ester (PMA)-induced NADPH oxidase activity and promote ROS production [23]. Therefore, the enhancement in the respiratory burst of neutrophils in the peripheral blood after EDTA injection may be caused by the high expression of the S100 family genes, which improves the sensitivity of neutrophils. It has also been shown that S100A8 and S100A9 can inhibit the oxidative metabolism of neutrophils in vitro, and adenosine metabolites can participate in the antioxidant effect of S100A8 and S100A9 [24,25]. In our preliminary experiments, changes in the adenylate metabolites of neutrophils after EDTA injection were also observed [11], which also provides further support for the involvement of adenosine metabolites and S100A8/A9 in the neutrophil respiratory burst. The role of the S100 family in inflammation and nutritional immunity has been discussed in previous studies and will not be repeated here [12].

In addition, the activity of zinc/copper superoxide dismutase (CuZn-SOD) increased significantly after EDTA injection. Superoxide dismutase (SOD) plays an irreplaceable role in the oxidation and antioxidation balance of the body [26]. It can remove superoxide anion (O2−) from cells, thereby preventing the generation of more dangerous hydroxyl radicals [27]. Cu is an essential cofactor for the active reaction of CuZn-SOD [20,28], whereas Zn does not directly react with superoxide anion radicals, but only plays a role in stabilizing the surrounding environment of the active center [28,29]. The significant increase in the serum Cu level after EDTA injection in the previous study may be related to an enhancement in CuZn-SOD activity. On the other hand, in addition to the effect of antioxidative stress, SOD also plays a vital role in the resolution of inflammation [30]. Therefore, from the perspective of feedback regulation, the enhancement in CuZn-SOD activity may serve to alleviate the inflammation caused by the injection of EDTA. In summary, the inflammatory response caused by the mineral element homeostasis imbalance in sheep may be related to the changes in neutrophil metabolism and gene transcription levels, which cause fluctuations in serum inflammation and antioxidant indicators and increase the respiratory burst of neutrophils.

## 5. Conclusions

Mineral element homeostasis imbalance in sheep, induced by an intravenous injection of EDTA, can enhance the respiratory burst of peripheral blood neutrophils, increase the level of IL-6 in serum and increase the risk of oxidative stress injury in sheep, which may be related to the increased expression of the S100 family of genes. The imbalance of serum mineral element homeostasis has adverse effects on the kidney health of animals, which may be related to a decrease in zinc ions. The imbalance of serum mineral element homeostasis can enhance the activity of CuZn-SOD in serum, which may be related to the negative feedback regulation of the inflammatory response. The fluctuation in serum-related indicators and the enhancement in the neutrophil respiratory burst caused by changes in metabolism and gene transcription levels will eventually affect the normal functioning of neutrophils in the inflammatory response.

## Figures and Tables

**Table 1:** Routine blood test results.

Item	Pre-Operation	Post-Operation
HCT/%	33.0 ± 1.0	31.0 ± 3.0
HGB/(g/dL)	10.73 ± 0.15	10.53 ± 1.12
MCHC/(g/dL)	32.60 ± 0.53	33.80 ± 0.10
WBC/(10^9^/L)	9.57 ± 1.04	10.33 ± 1.10
GRANS/(10^9^/L)	5.70 ± 1.25	7.70 ± 1.78
L/M	9.59 ± 0.04	9.41 ± 0.12

**Table 2:** Blood gas and electrolyte measurements of sheep in the control and EDTA groups.

Item	Control Group	EDTA Group
pH	7.40 ± 0.42	7.44 ± 0.45
PCO2	36.00 ± 4.27	35.88 ± 4.89
HCO3^−^	21.32 ± 2.77	22.70 ± 3.54
tCO2	22.31 ± 2.88	23.80 ± 3.64
Na^+^	143.60 ± 1.90	137.13 ± 3.64 **
K^+^	3.83 ± 0.35	2.35 ± 0.43 **
Cl^−^	108.10 ± 2.42	103.50 ± 2.62 **
Ca^2+^	1.07 ± 0.09	1.36 ± 0.36

** *p* < 0.01.

## Data Availability

The data presented in this study are available on request from the corresponding author.
